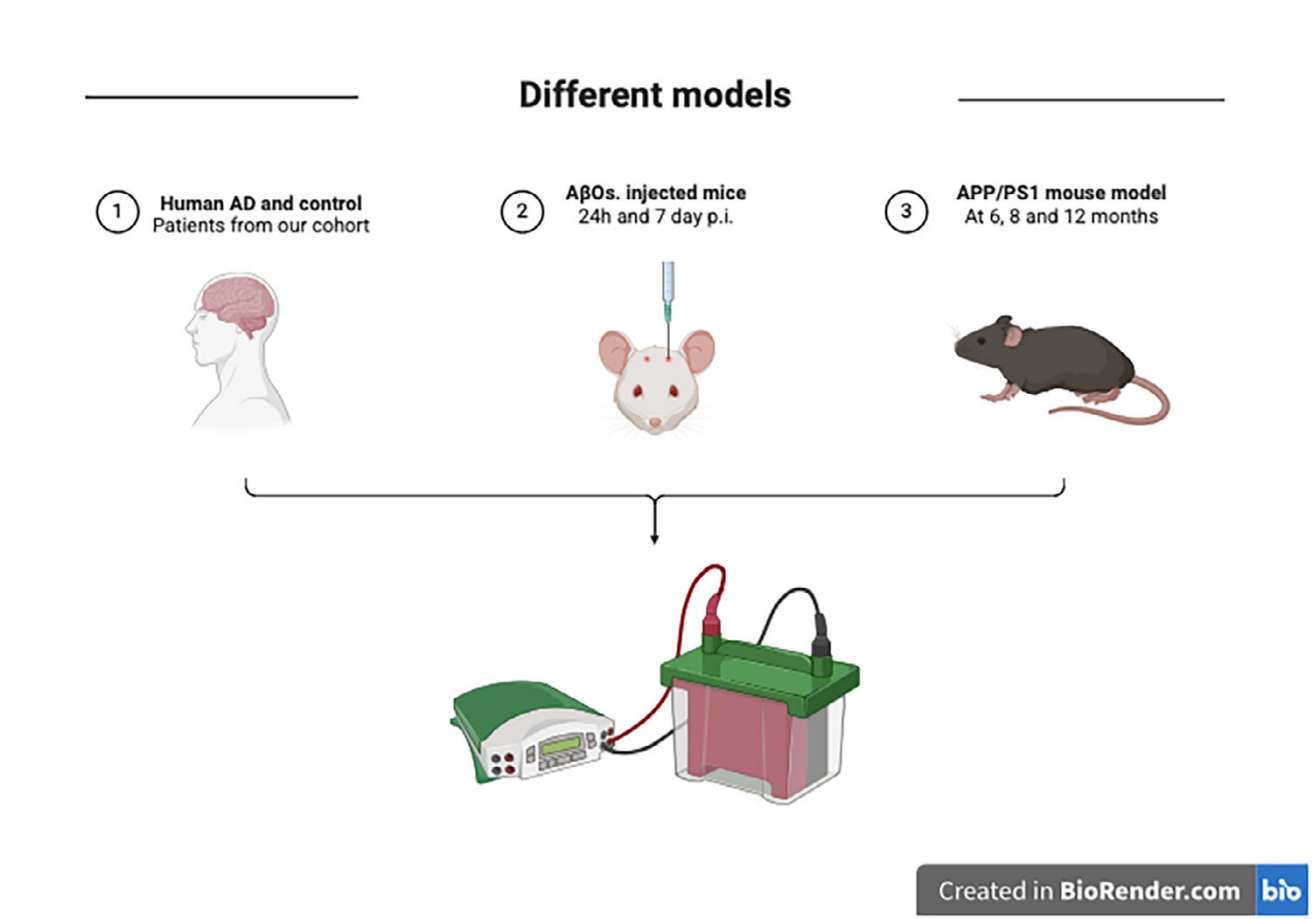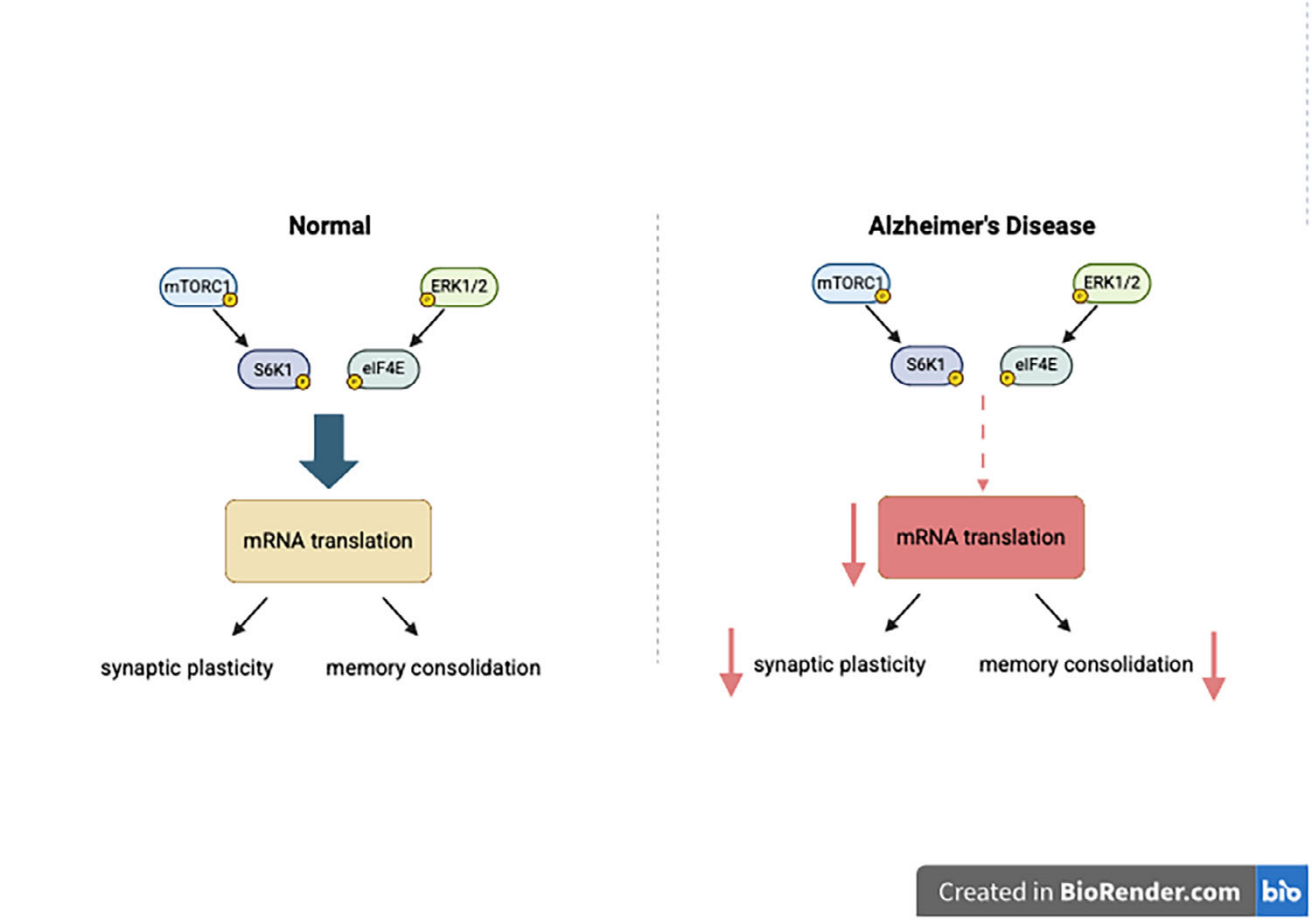# Exploring mTOR and protein synthesis regulation in Alzheimer's disease: insights from human and animal models

**DOI:** 10.1002/alz70855_099107

**Published:** 2025-12-23

**Authors:** Rubens L Soares‐Neto, Felipe C. Ribeiro, Joao D Calixtro, Danielle Cozachenco Ferreira, Sergio T. Ferreira

**Affiliations:** ^1^ Institute of Biophysics Carlos Chagas Filho, Federal University of Rio de Janeiro, Rio de Janeiro, Rio de Janeiro, Brazil; ^2^ Federal University of Rio de Janeiro, Rio de Janeiro, Rio de Janeiro, Brazil; ^3^ Federal University of Rio de Janeiro, Rio de Janeiro, Brazil; ^4^ Institute of Biophysics, Federal University of Rio de Janeiro, Rio de Janeiro, Brazil; ^5^ Federal University of Rio de Janeiro, Rio de Janeiro, RJ, Brazil; ^6^ D'Or Institute for Research and Education, Rio de Janeiro, RJ, Brazil

## Abstract

**Background:**

mTOR is a key protein in the integration of metabolic and energetic signals, regulating cellular processes such as protein synthesis, which is essential for memory consolidation and synaptic plasticity. Our group has shown that drugs modulating mRNA translation, such as HNK, can reverse cognitive deficits in AD models. This study aims to expand the understanding of mTOR regulation and protein synthesis in AD patients and models.

**Method:**

We used Western Blotting (WB) to analyze cortical and hippocampal samples from AD patients and controls, as well as animal models at different ages, including APP/PS1 (4‐6 months; 8‐10 months; 12‐14 months) and mice injected with beta‐amyloid oligomers (24 hours and 7 days after injection). Proteins related to the mTOR pathway, including total and phosphorylated mTOR, S6K and ERK, were then evaluated.

**Results:**

In animal models, no differences were observed in phosphorylated mTOR levels relative to total mTOR. In AD patients, a trend toward reduced phosphorylated ERK relative to total ERK was identified.

**Conclusion:**

These findings highlight the importance of understanding specific alterations in different experimental systems to elucidate potential therapeutic targets of AD.